# Changes in mental health during three waves of the COVID-19 pandemic: a repeated cross-sectional study among Polish university students

**DOI:** 10.1186/s12888-021-03615-2

**Published:** 2021-12-15

**Authors:** Aleksandra M. Rogowska, Dominika Ochnik, Cezary Kuśnierz, Karolina Chilicka, Monika Jakubiak, Maria Paradowska, Luiza Głazowska, Dawid Bojarski, Julia Fijołek, Marcin Podolak, Maciej Tomasiewicz, Dominika Nowicka, Marek Kawka, Maksymilian Grabarczyk, Zuzanna Babińska

**Affiliations:** 1 Institute of Psychology, University of Opole, Opole, Poland; 2 Faculty of Medicine, University of Technology, Katowice, Poland; 3 Faculty of Physical Education and Physiotherapy, Opole University of Technology, Opole, Poland; 4 Institute of Health Sciences, University of Opole, Opole, Poland; 5 Faculty of Economics, Maria Curie-Sklodowska University in Lublin, Lublin, Poland; 6 Faculty of Psychology and Cognitive studies, Adam Mickiewicz University in Poznań, Poznań, Poland; 7 Institute of Psychology, University of Lodz, Łódź, Poland; 8 Faculty of Medicine, Poznan University of Medical Sciences, Poznań, Poland; 9 Faculty of Medicine, Wroclaw Medical University, Wrocław, Poland; 10 Faculty of History, University of Warsaw, Warszawa, Poland; 11 Faculty of “Artes Liberales”, University of Warsaw, Warszawa, Poland; 12 Institute of the Middle and Far East, Faculty of International and Political Studies, Jagiellonian University, Kraków, Poland

**Keywords:** Anxiety, Life satisfaction, Logistic regression, Perceived stress, Physical health, Prevalence

## Abstract

**Background:**

Research indicates that mental health worsened during the Coronavirus crisis, in particular among women and university students. However, few longitudinal studies have so far investigated the changes in mental health outcomes across three subsequent waves of the COVID-19 pandemic. Therefore, this study aims to examine changes in mental health among university students.

**Methods:**

A total of 1,961university students from Poland, at mean age 23.23 years (*SD* = 3.16, 57.47% of women) were included in this repeated cross-sectional study across three waves of the COVID-19 pandemic: W1 (*n* = 657), W2 (*n* = 654), and W3 (*n* = 650). They completed the online survey with the Generalized Anxiety Disorder (GAD-7), Perceived Stress Scale (PSS-10), General Self-Rated Health (GSRH), and Satisfaction with Life Scale (SWLS), as well as sociodemographic variables.

**Results:**

The prevalence of people at high risk of anxiety and perceived stress, poorer physical health, and low life satisfaction changed significantly across three waves of the COVID-19 pandemic. The results of the two-way ANOVA showed that both the wave (W1<W2<W3) and gender (men<women) had a significant impact on the level of anxiety. Statistically significant changes in perceived stress were found between pandemic waves (W1>W2, W1>W3), and genders (men<women). Self-reported physical health significantly deteriorated in W3 compared to W1 and W2 (W1>W3, W2>W3), and was significantly worse in women than in men. The level of life satisfaction also decreased significantly in W3 (W1>W3, W2>W3), but did not differ between men and women. High GAD risk was presented two times more frequently among women and people who subjectively assessed their health as poor, three times more likely in participants dissatisfied with their lives, and seven times more probably in persons with high-stress levels.

**Conclusions:**

The results of this study consistently indicate (using parametric and non-parametric statistical analysis) that there are significant differences in mental health problems across three waves of the COVID-19 pandemic. It suggests that pandemic waves should be considered in future review studies and meta-analyses. Furthermore, these findings indicate a potential role for prevention and intervention programs aimed at alleviating life satisfaction and subjective assessment of health and improving coping skills to reduce stress and anxiety.

## Introduction

The Coronavirus disease (COVID-19) spread globally in March 2020. From the start of the pandemic to October 4, 2021, the coronavirus infected 235.08 million people worldwide, and 5,009,716 of whom died [1]. Although a small percent of the population was infected or died with the Coronavirus, numerous restrictions and lockdowns, entering subsequently in various countries on the world, changed significantly economic status and lifestyle of many populations, causing high stress and anxiety [2–4]. As a consequence, remote learning or work, as well as limitation of physical activity, forced unexpected changes in daily life routines [5–10]. The COVID-19 pandemic impacted the mental health of people around the world [11–19], including a high prevalence of depression, anxiety, insomnia, posttraumatic stress disorder (PTSD), and psychological distress.

A systematic review and meta-analysis [14] conducted globally (including 32 different countries and 398,771 participants) showed the pooled prevalence of anxiety in 26.9% of people, 36.5% for perceived stress, 50.0% for psychological distress, 30.7% for somatic symptoms and 28.6% for low wellbeing. However, the prevalence of mental health problems may be different in particular countries. For example, considering recent systematic reviews and meta-analytic studies, the prevalence of perceived stress ranges between 13.29% [12], through 29.6% [17], 36.5% [14], 37.54% [13], to 48.1% [11] during the COVID-19 pandemic. The anxiety prevalence during the pandemic was rated as 15.15% [12], 21.8% [11], 25% [16], 26.9% [14], 31.9% [17], or 38.12% [13].

The prevalence of mental health problems may be determined by geographic region, period of data collection, the poverty impacts of COVID-19, preparedness of countries to respond, economic vulnerabilities, diagnostic criteria of the given toll for measurement of mental health issues, education level, living arrangement, as well as other multiple epidemics and psychosocial factors related to cross-cultural differences [8, 13, 14]. In particular, female gender was found as a risk factor of mental health problems in numerous studies [8, 11, 20–30]. In addition, the decreased levels of physical activity during the lockdown [31], as well as high screen time per day (e.g., using computers or tablets, watching TV) worsened mental health [32] Also, university students may be more vulnerable to mental health issues than the general population [8, 20, 23–27, 33–39]. A systematic review and meta-analysis by Deng et al. [8] showed that anxiety symptoms are higher among university students during the COVID-19 pandemic compared to pre-pandemic prevalence in similar populations. A nationwide cross-sectional survey study of 821,218 college students, conducted in China in February 2020 [37], found mental health problems among 45% of participants. Prevalence rates of stress among Chinese students was 34.9%, while anxiety symptoms were 11.0% (using the GAD-7). Wathelet et al. [28] performed research among 69,054 university students living in France during the first COVID-19 quarantine (data were collected from April 17 to May 4, 2020). A high percentage of participants (42.8%, *n* = 29 564) reported at least one mental health problem among perceived stress, severe distress, anxiety, and depression. The high level of perceived stress experienced 24.7% (17 093 students), while a high level of anxiety was 27.5% (18 970 students). Deng et al. [8] found the pooled prevalence of anxiety symptoms as 32% among the university student’s population (*N* = 1,441,828).

The present study will examine the prevalence of anxiety, perceived stress, physical health, and life satisfaction in the Polish sample of university students. Most of the available studies were performed during the first wave of the COVID-19 pandemic. However, little is known whether the differences in the prevalence of mental health problems vary across the following waves of the COVID-19 pandemic. This study aims to examine changes in the mental health of university students, comparing three samples that participated in observational cross-sectional studies during three waves of the COVID-19 pandemic, respectively. Taking into account the dynamic of the spread of the Coronavirus disease and corresponding level of restrictions and lockdown duration, as well as human resilience and adaptation to changes, we expect that the levels of anxiety, perceived stress, subjective assessment of physical health, and life satisfaction, will differ across three waves of the pandemic. Also, gender differences are expected in the study, with a higher risk of mental health problems among women than in men. To find the best predictors of GAD risk, we will examine associations between anxiety and pandemic waves, gender, perceived stress, subjective assessment of physical health, and life satisfaction.

## Methods

### Study design, data collection and participants

**Fig. 1:**
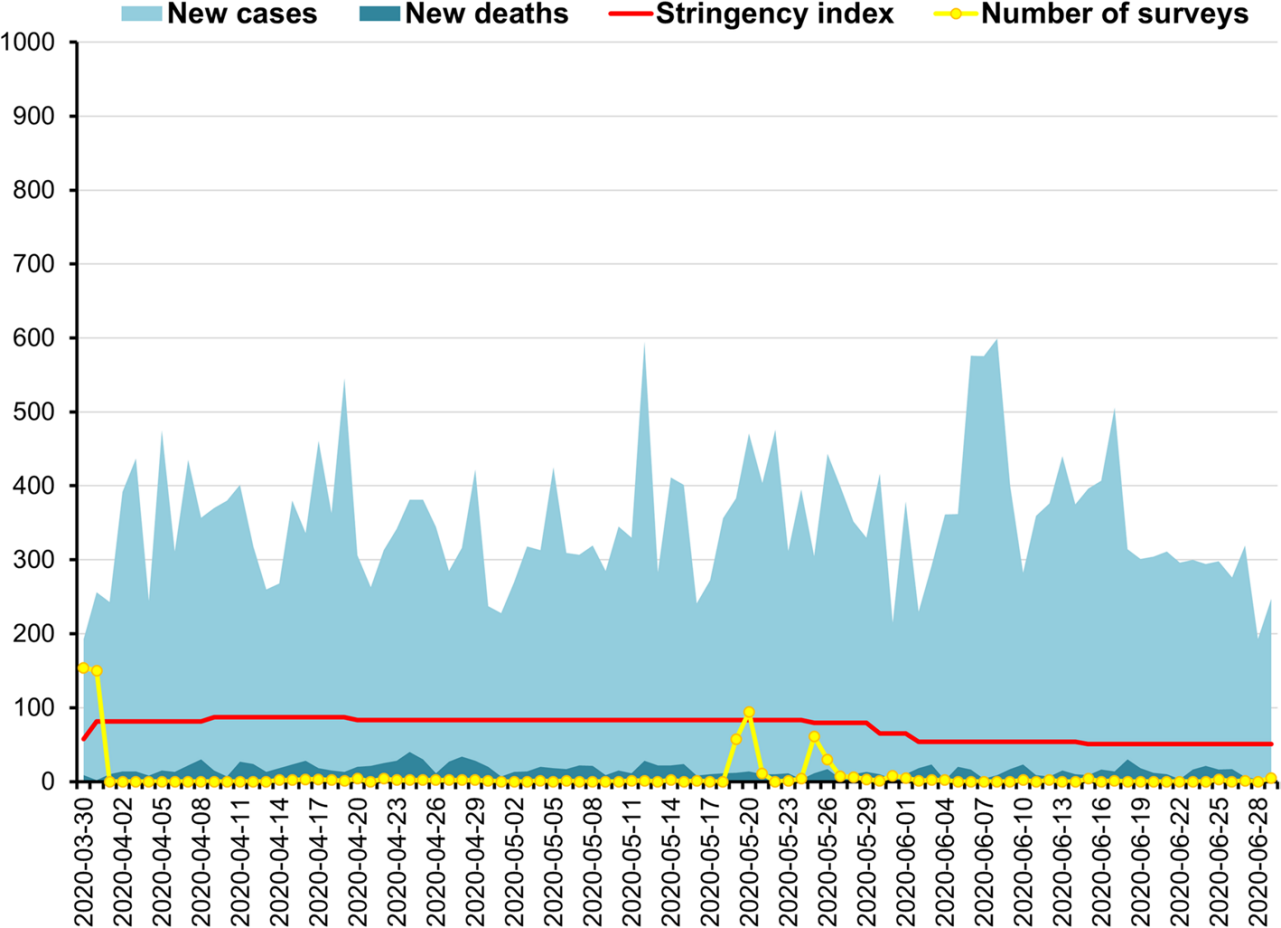
The first wave of the COVID-19 pandemic in Poland, between 30th March 2020 and 29th June 2020: New cases of Coronavirus ranged 193 - 599 daily (*M* = 351.00, *SD* = 86.95); New deaths from Coronavirus ranged 2 - 40 daily (*M* = 15.46, *SD* = 7.89); Stringency index ranged 50.93 - 87.04 (*M* = 73.04, *SD* = 14.59); Number of surveys ranged 0 - 154 daily (*M* = 7.15, *SD* = 25.40). Source of new cases, new deaths, and stringency index during the COVID-19 pandemic in Poland: Johns Hopkins University Center for Systems Science and Engineering (CSSE) COVID-19 Data [40]

**Fig. 2:**
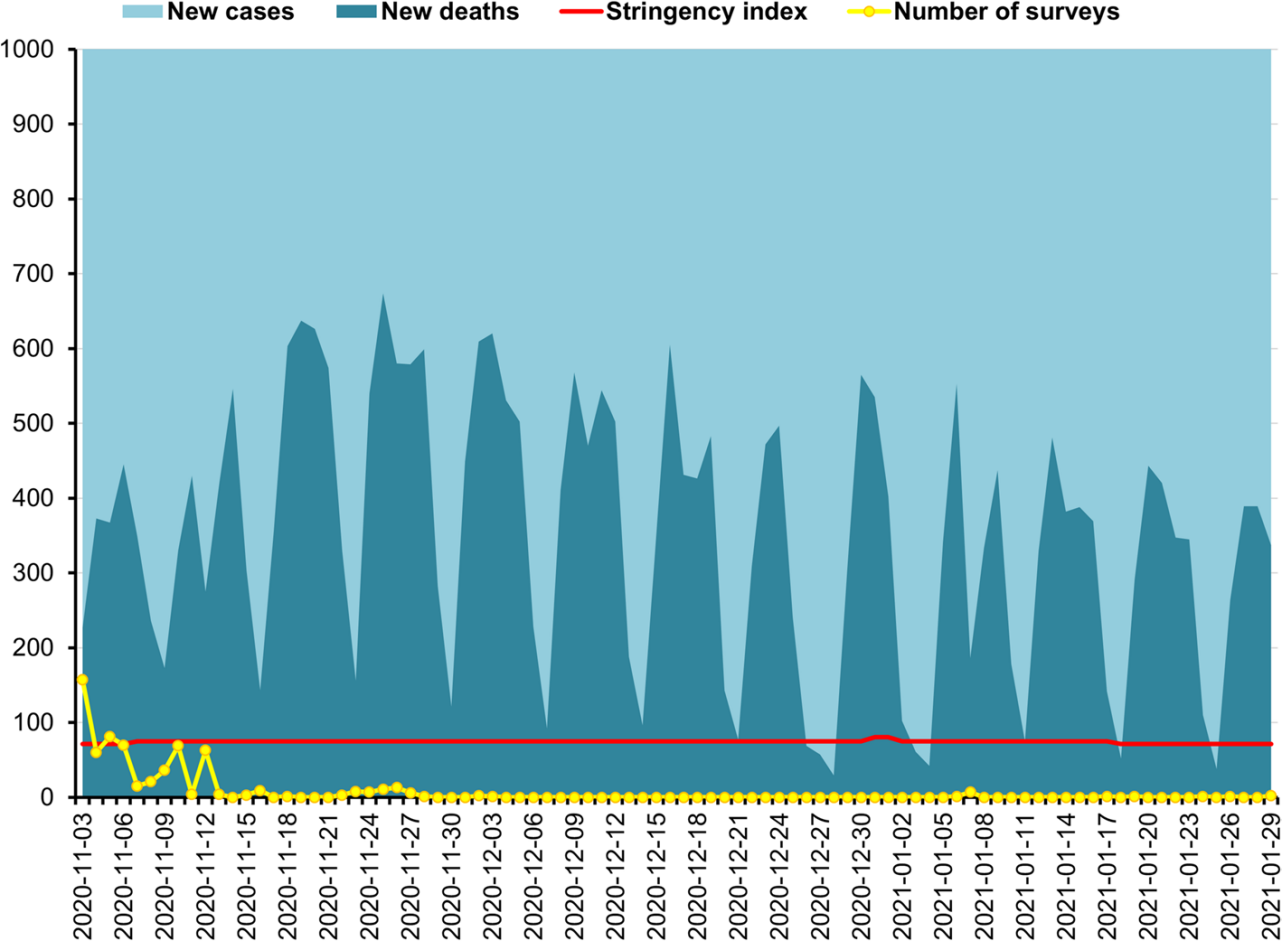
The second wave of the COVID-19 pandemic in Poland, between 10th April and 12 June 2021: New cases of Coronavirus ranged 192 - 24,892 daily (*M* = 5376.35, *SD* = 6179.14); New deaths from Coronavirus ranged 7 - 804 daily (*M* = 263.78, *SD* = 233.22); Stringency index ranged 53.7 - 75.93 (*M* = 65.09, *SD* = 7.41); Number of surveys ranged 0 - 134 daily (*M* = 10.03, *SD* = 21.30). Source of new cases, new deaths, and stringency index during the COVID-19 pandemic in Poland: Johns Hopkins University Center for Systems Science and Engineering (CSSE) COVID-19 Data [40]

A cross-sectional repeated study was performed three times in Poland. The first study was conducted during the first wave of the COVID-19 pandemic (W1), between 30th March and 29th June 2020, the second (W2) between 3rd November 2020 and 29th January 2021, and the third (W3) between 10th April and 12 June 2021. In Poland, the pandemic started on January 21. Average number of new confirmed cases per day during the data collection period was *M* = 351.00, *SD* = 86.95 at W1 (Fig. 1), during W2 was *M* = 12,583.30, *SD* = 7,273.55 (Fig. 2), while at W3 was *M* = 5,456.83, *SD* = 6,193.56 (Fig. 2). During the same period, the average daily number of new deaths was *M* = 15.46, *SD* = 7.89 at W1, during W2 was *M* = 351.19, *SD* = 176.88, and at W3 was *M* = 267.73, *SD* = 232.86 [40]. The pick of W2 was on November 8, 2020, and at W3 was on April 1, 2021, while wave pick was not observed at W1 in Poland.

The response to COVID-19 can be reflected in the stringency index (SI) of the COVID-19 [41]. The SI is a composite measure of restriction level, based on nine response indicators, including school closures, workplace closures, and travel bans, rescaled to a value from 0 to 100 (100 is the strictest). We calculated the mean (*M*) and standard deviation (*SD*) of restriction measured in SI during the given period, whereas absolute change (AC) and relative change (RC) were presented by Hale et al. [41]. During W1, between 30th March (SI = 57.41) and 29th June 2020 (SI = 50.93) SI ranged from 50.93 to 83.33, *M* = 73.04, *SD* = 14.59, AC = -6.48, RC = -11%. When research were performed at W2 between 3rd November 2020 (SI = 71.3) and 29th January 2021 (SI = 71.3), SI ranged between 71.3 and 80.56, *M* = 74.45, *SD* = 1.71, AC = 0, RC = 0%. During W3, SI ranged between 75.93 (on 10th April 2021) and 53.70 (on 12 June 2021), *M* = 63.26, *SD* = 7.33, AC = -22.23, RC = -0.29%).

A priori test for required sample size was determined using G*Power software [42]. With a medium effect size *W* = 0.30, an alpha-level of *p* = 0.001, a power of 0.99, and *df* = 2, an analysis for the χ^2^ contingency table resulted in a minimal sample size of *n* = 392 (non centrality parameter λ = 35.28, critical χ^2^ = 13.82). A minimal sample size for two-way ANOVA was calculates as 149, with medium effect size η²_*p*_ = 0.05, *p* = 0.001, a power 0.99, and df = 2 for 3 groups and two covariates (non centrality parameter λ = 37.25, critical *F* = 7.25). To avoid missing data, the survey required participants to complete all answers on each page. Therefore, participants filling in the questionnaire via Google Form did not omit any answers, except some demographic data with the option “other answer.“

Students were recruited from the following universities: Jagiellonian University, Lodz University of Technology, Maria Curie-Sklodowska University in Lublin, Nicolaus Copernicus University in Toruń, Opole University of Technology, Poznan University of Medical Sciences, SWPS University, University of Lodz, University of Opole, the University of Technology in Katowice, University of Warsaw, University of Zielona Gora, Wroclaw Medical University. The online survey was created in Google Forms and distributed through a personal e-mailing list, as well as banners with an invitation to participate in the study were disseminated on e-learning platforms (such as Moodle or Teams), and various groups of students at social media (such as Facebook and Instagram).

To minimalize sources of bias, we collected a diverse student sample in terms of its key characteristics: type of university (humanistic university, university of technology, medical university, fine art university), field and major of study, study level, year, and type. The eligibility criterion was being a college or university student and had at least 18 years old. All respondents were eligible for inclusion in the research and confirmed their student status by answering their current field, major, level, year, and type of study.

### Ethical consideration

The local Institutional Research Board (IRB) at the University of Opole approved the study protocol (1/2020). Students were assured about the anonymity and confidentiality of the survey, and they were informed that they could pull out of the study whenever they wanted. Informed consent was obtained from all participants since information about the study and informed consent was included in the first part of the questionnaire. The study was conducted according to the 1964 Helsinki Declaration and its later amendments or comparable ethical standards (like the APA ethical standards). No compensation was offered to students or researchers as an incentive to participate. The authors received no specific funding for this work.

### Measures

#### Anxiety

Anxiety risk was assessed using the 7-items generalized anxiety disorder (GAD-7) scale [43]. The GAD-7 is a brief self-reported screen toll to measure anxiety symptoms understand as a persistent and excessive worry about various issues, following the Diagnostic and Statistical Manual of Mental Disorders, fifth edition (DSM-5) criteria. Participant rates on a 4-point Likert scale (0 = *Not at all*, 1 = *Several days*, 2 = *More than half the days*, and 3 = *Nearly every day*), how often he/she experienced anxiety symptoms during the last two weeks. Higher scores indicate higher general anxiety disorder (GAD) risk. The Cronbach’s α for the GAD-7 in this study was 0.93.

#### Perceived stress

Psychological stress was measured using the Perceived Stress Scale (PSS-10). The PSS was developed by Cohen et al. [44], as a self-report ten-item questionnaire, with a 5-point Likert scale (ranging from 0 = *Never* to 4 = *Very often*). Participant indicates how often he/she experienced a given type of behavior during the past month. Total scores range between 0 and 40, and higher scores indicate higher levels of perceived stress. The reliability coefficient Cronbach’s α = 0.59.

#### Physical health

A subjective assessment of physical health was reached using two single-item questions of the General Self-Rated Health (GSRH) [45, 46]. The GSRH is a shorter alternative to the standard general health survey (SF-12 V). The first question, GSRH 1, concerned overall physical health (“In general, would you say your health is…?“), while the second, GSRH 2, is a comparison of self-health with other people (i.e., “Compared to others your age, would you say your health is…?“). Participants rated on a 5-point Likert scale (1 = *Excellent*, 2 = *Very Good*, 3 = *Good*, 4 = *Fair*, and 5 = *Poor*) how they subjectively assess their health. A higher score indicates a worse self-rated health status. DeSalvo et al. [45] showed that high scores of the GSRH (poor health) have a strong association with mortality. In the study, the internal consistency of GSRH (Cronbach’s α) was 0.87.

#### Life satisfaction

The Satisfaction with Life Scale (SWLS) is a short 5-item measure of global cognitive judgments regarding satisfaction with one’s life [47, 48]. Respondents indicate how much they agree or disagree with the given sentence (from 7 = *Strongly agree*, to 1 = *Strongly disagree*). Total scores ranged from 5 to 35, and high scores indicate high satisfaction with life. In the current study, the reliability coefficient Cronbach’s α was 0.85.

#### Demographic data

Demographic questionnaire included questions about age (number of years), gender (female, male), place of residence (village, town, city, agglomeration), the field of study, study major, level of study (3-years Bachelor, 2-years Master, 5-years Master, doctoral), study grade (from 1th – to 5th study year), and type of study (full-time, part-time).

### Statistical analysis

First, a series of contingency Table (3 × 2) was created for association between three waves of the COVID-19 pandemic (W1. W2, and W3) and GAD considered as categorical variable (dummy coding: 0 = no anxiety risk, for the GAD-7 scores < 10; 1 = anxiety risk if the GAD-7 score ≥ 10), perceived stress (0 = low stress, PSS-10 < 24; 1 = high stress, PSS ≥ 24), physical health (0 = good health status, GSRH = 1, 2, or 3; 1 = poor health status, GSRH = 4 or 5); life satisfaction (0 = satisfied, SWLS > 19; 1 = unsatisfied SWLS ≤ 19). A Pearson’s χ^2^ test was performed, with Cramer’s *V* for effect size assessment. Also, a 2 × 2 contingency tables were performed with gender (Women, Men) and such mental health categorical variables as GAD risk (No anxiety risk, Anxiety risk), perceived stress (Low stress, High stress), physical health (Good health status, Poor health status), and life satisfaction (Satisfied, Unsatisfied). A Pearson’s χ^2^ test was conducted to examine associations between gender and anxiety, with an ϕ coefficient to calculate an effect size.

The parametric properties were tested using a range of scores, mean (*M*), standard deviation (*SD*), median, skewness, and kurtosis, for all continuous variables, including anxiety (GAD-7), perceived stress (PSS-10), physical health (GSRH 1 and GSRH 2), and life satisfaction (SWLS). Since good properties were found, a series of two-way ANOVAs were performed to examine the effect of three waves of the COVID-19 pandemic (W1, W2, and W2) and gender (Women, Men) on anxiety, perceived stress, physical health, and life satisfaction. Effect sizes were calculated using partial eta-squared statistic (η_*p*_^2^). All above mentioned statistical analyses were performed using JASP ver. 0.14.1 software for Windows [49].

Finally, the logistic regression was performed to examine predictors of GAD risk in the total sample of university students, among such categorical (binary) variables as gender, pandemic wave, perceived stress, subjective assessment of physical health, and life satisfaction. We used enter method of variables selection, which means that all variables in a block were entered in a single step. The IBM SPSS Statistics for Windows, ver. 26.0 software was used for calculating a logistic regression.

## Results

### Demographic characteristic

Initially, data collection included 2015 people, but 13 students refused to participate in the study at W1 (*N* = 670, 1.94%), 17 (*N* = 671, 2.53%) at W2, and 24 (*N* = 674, 3.56%) at W3. The final total sample included 1961 university students. The response rate was 97.32%. The sample size exceeds the required sample size, which improves the power of the statistical tests. The average time to complete the survey was 23 min (*M* = 23.26; *SD* = 44.03).

The total sample of 1,961 university students consisted of three different cohorts recruited during three following waves of the COVID-19 pandemic: Sample 1 = 657 people at W1, Sample 2 = 654 at W2, and Sample 3 = 650 at W3. The age of participants ranged between 19 and 49 years, with mean age of 23 (*M* = 23.23, *SD* = 3.16) in the total sample, and also in Sample 1 (range 19-48, *M* = 23.68, *SD* = 2.66), Sample 2 (range 19-46, *M* = 22.72, *SD* = 3.15), and Sample 3 (range 19-49, *M* = 23.31, *SD* = 3.51). Table 1 shows the demographic characteristic of three samples as well as the total sample. Among participants prevailed women than men (19 people preferred not to answer the gender question), those living in village or towns (than in city or agglomeration), a Bachelor’s degree (in comparison to Master and Doctoral levels), studying in the first year, and at full-time study. The Pearson’s χ^2^ independence test showed, that samples differ significantly in gender (Cramer’s V = 0.08), place of residence (Cramer’s V = 0.24), study level (Cramer’s V = 0.022), study year (Cramer’s V = 0.16), and study type (Cramer’s V = 0.20).

**Table 1:** Demographic characteristics of the sample

Variable		Total Sample (*N* = 1961)		Sample 1 (*n* = 657)			Sample 2 (*n* = 654)			Sample 3 *(n* = 650)				
		*n*	%	*n*	%	*n*		%	*n*		%	χ^2^	*df*	*V*
Gender												13.71**	2	0.08
	Women	1120	57.47	397	60.43	340		51.99	390		61.51			
	Men	815	41.56	260	39.57	311		48.01	244		38.49			
Place of residence												221.32***	6	0.24
	Village	764	38.96	309	47.03	288		44.04	167		25.69			
	Town	701	35.75	233	35.46	269		41.13	199		30.62			
	City	403	20.55	103	15.68	92		14.07	208		32.00			
	Agglometation	91	4.64	10	1.52	5		0.76	76		11.69			
Study level												176.18***	6	0.22
	Three-years Bachelor	1151	58.69	371	56.47	455		69.57	325		50.00			
	Two-years Master	306	15.60	162	24.66	89		13.61	55		8.46			
	Five-years Master	493	25.15	119	18.11	109		16.67	265		40.77			
	Doctoral	11	0.56	5	0.76	1		0.15	5		0.77			
Study year												93.91***	8	0.16
	1th year	650	33.15	237	36.07	238		36.39	175		26.92			
	2nd year	470	23.97	147	22.37	209		31.96	114		17.54			
	3rd year	484	24.68	172	26.18	111		16.97	201		30.92			
	4th year	243	12.39	61	9.28	73		11.16	109		16.77			
	5th year	114	5.81	40	6.09	23		3.52	51		7.85			
Study type												77.77***	2	0.20
	full-time	1697	86.54	595	90.56	602		92.05	500		76.92			
	part-time	264	13.46	62	9.44	52		7.95	150		23.08			

*p < 0.05, **p < 0.01, ***p < 0.001

### Prevalence of mental health problems across three waves of the pandemic

**Fig. 3:**
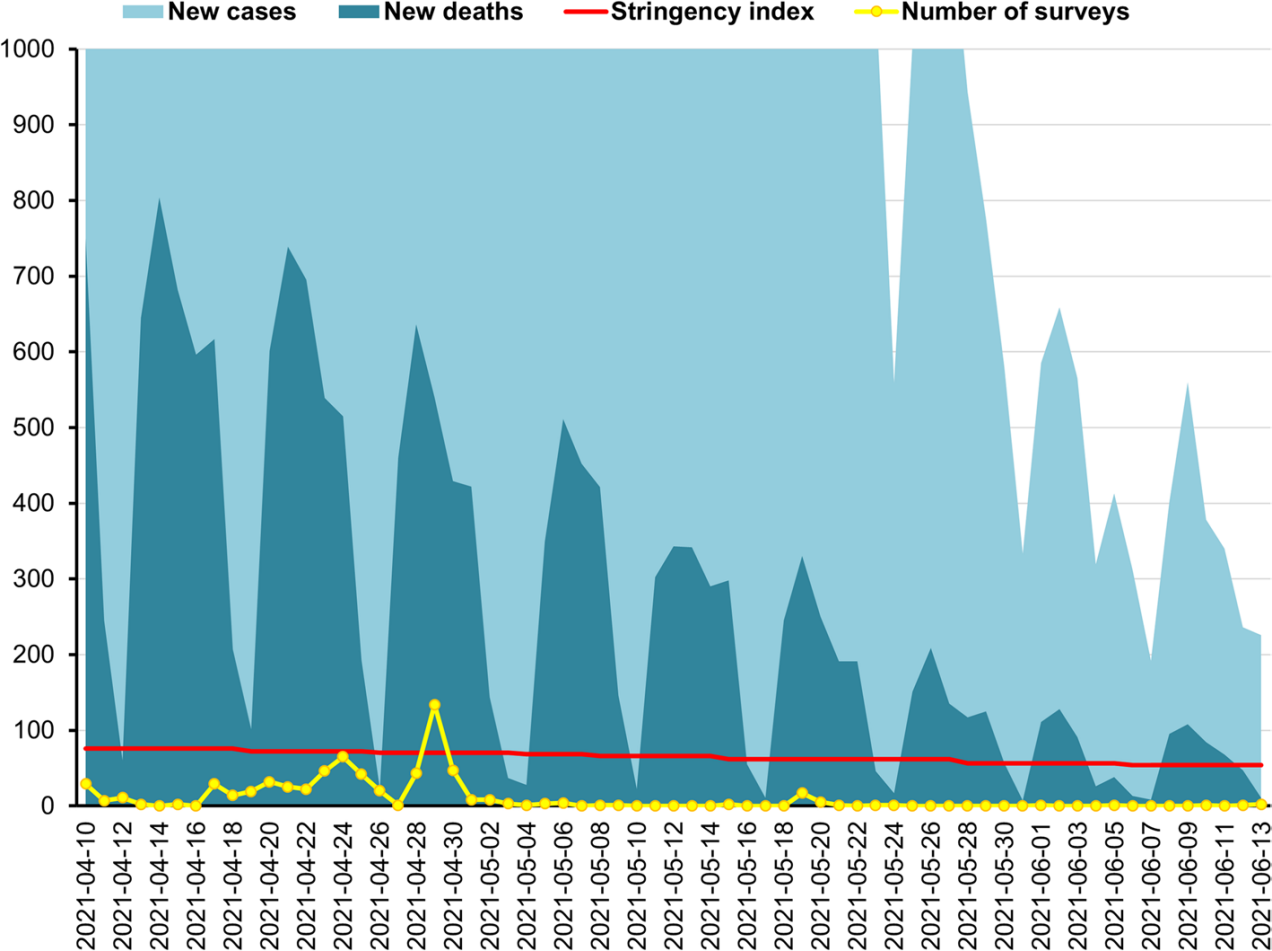
The third wave of the COVID-19 pandemic in Poland, between 10th April and 12 June 2021: New cases of Coronavirus ranged 192 - 24892 daily (* M* = 5376.35, *SD* = 6179.14); New deaths from Coronavirus ranged 7 - 804 daily (*M* = 263.78, *SD* = 233.22); Stringency index ranged 53.7 - 75.93 (*M* = 65.09, SD = 7.41); Number of surveys ranged 0 - 134 daily (*M* = 10.03, *SD* = 21.30). Source of new cases, new deaths, and stringency index during the COVID-19 pandemic in Poland: Johns Hopkins University Center for Systems Science and Engineering (CSSE) COVID-19 Data [40]

Frequency of people with anxiety risk during three waves of the COVID-19 pandemic is shown in Fig. 3. The GAD risk (scores of GAD-7 ≥ 10) was found in 753 people, which is 38.4% of the total sample (*N* = 1961). The differences in GAD risk across three waves of the COVID-19 pandemic W1 (*n* = 252, 38.36%), W2 (*n* = 200, 30.58%), and W3 (*n* = 301, 46.31%), were significant but effect size was weak, χ^2^(2) = 34.09, *p* < 0.001, Cramer’s *V* = 0.13. A 2 × 2 contingency table was created for gender, to examine its association with GAD risk. Anxiety risk prevailed in women (*n* = 513, 45.52%) than in men (*n* = 228, 27.98%), with weak effect size, χ^2^(1) = 61.69, *p* < 0.001, ϕ = 0.18.

A high level of perceived stress (PSS-10 ≥ 24) was presented in 1,213 university students (61.86%), including 530 people at W1 (80.67%), 324 at W2 (49.54%), and 359 at W3 (55.23%). The differences between three waves were significant, with moderate effect size, χ^2^(2) = 152.69, *p* < 0.001, Cramer’s *V* = 0.28. Significantly more women (*n* = 780, 62.21% of the women sample) than men (*n* = 421, 51.66% of men sample) experienced a high level of perceived stress, but these differences were weak, χ^2^(1) = 61.76, *p* < 0.001, ϕ = 0.18.

Physical health was considered at both items GSRH 1 and GSRH 2. Among university students sample, 199 participants (10.15%) self reported their health as fair or poor (GSRH 1), while 322 persons (16.42%) as worse in comparison to other people at the same age (GSRH 2). The differences in GSRH 1 between W1 (*n* = 43, 6.54%), W2 (*n* = 46, 7.03%), and W3 (*n* = 110, 16.92%) were significant but weak, χ^2^(2) = 49.03, *p* < 0.001, Cramer’s *V* = 0.16. Similarly, differences in GSRH 2 between W1 (*n* = 81, 12.33%), W2 (*n* = 70, 10.7%), and W3 (*n* = 171, 26.31%) were significant but effect size was also weak, χ^2^(2) = 69.89, *p* < 0.001, Cramer’s *V* = 0.19. Gender was not associated with physical health, since as many percent of women (*n* = 116, 10.29% of the women sample) as men (*n* = 77, 9.46% of the men sample) poorly rated their physical health, χ^2^(1) = 0.38, *p* = 0.54, ϕ = 0.01. However, when participants compared their health with others at the same age, significantly more women felt worse (*n* = 204, 18.10%) than men (*n* = 111, 13.62%), χ^2^(1) = 6.99, *p* < 0.001, ϕ = 0.06.

Among university students, 830 people was dissatisfied with their life (SWLS ≤ 19), which is 42.33% of the total sample (*N* = 1961). A statistically significant differences in life satisfaction was found between W1 (*n* = 246, 37.44%), W2 (*n* = 262, 40.06%), and W3 (*n* = 322, 49.54%), but the effect size was very small, χ^2^(2) = 21.64, *p* < 0.001, Cramer’s *V* = 0.11. Similar percent of women (*n* = 471, 41.79%) was unsatisfied with their life as percent of men (*n* = 347, 42.58%), so gender differences were not found, χ^2^(1) = 0.12, *p* = 0.73, ϕ = 0.00.

### Descriptive statistics for study variables

The preliminary analysis was performed to examine parametric properties of the variables considered as continuous. Descriptive statistics contained a range of scores, mean (*M*), standard deviation (*SD*), median, skewness, and kurtosis. Each variable, including anxiety (GAD-7), perceived stress (PSS-10), physical health (GSRH), and life satisfaction (SWLS), were assessed during W1, W2, and W3 (see Table 2 for more details). Skewness and kurtosis ranged between ±1, meaning that all variables demonstrate good properties, and parametric statistical tests can be used to further analysis.

**Table 2:** Descriptive statistics for study variables

Variable	Wave	Range	*M*	*SD*	Median	Skewn.	Kurtos.
GAD	W1	0-21	8.39	5.63	7	0.52	-0.67
	W2	0-21	7.32	5.64	7	0.59	-0.49
	W3	0-21	9.37	5.94	9	0.27	-0.97
PSS	W1	7-43	28.72	5.70	29	-0.17	-0.21
	W2	0-39	23.15	5.07	23	-0.50	1.31
	W3	0-40	23.53	4.76	24	-0.45	1.13
GSRH 1	W1	1-5	2.41	0.79	2	0.25	0.21
	W2	1-5	2.48	0.78	2	0.25	0.35
	W3	1-5	2.76	0.87	3	0.08	-0.01
GSRH 2	W1	1-5	2.52	0.87	3	0.13	-0.32
	W2	1-5	2.51	0.87	2	0.29	0.08
	W3	1-5	2.94	0.98	3	0.09	-0.30
SWLS	W1	5-35	21.2	6.21	22	-0.29	-0.42
	W2	5-35	20.85	6.09	21	-0.14	-0.38
	W3	5-35	19.78	7.00	20	0.05	-0.83

*Note.* GAD = general anxiety disorder; PSS = perceived stress scale; GSRH = general self-rated health; SWLS = satisfaction with life scale

### Changes in mental health during three waves of the COVID-19 pandemic

**Fig. 4:**
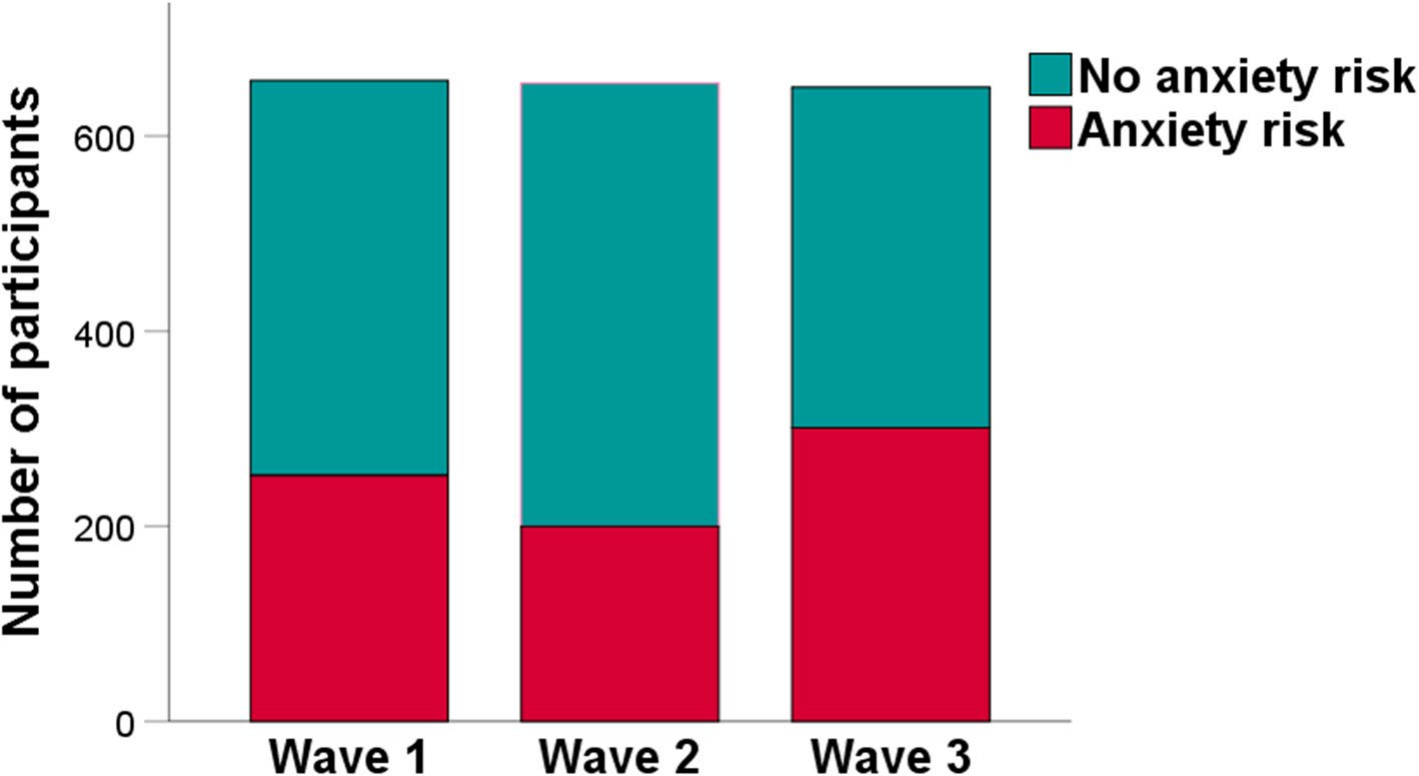
Frequency of people with anxiety risk during three waves of the COVID-19 pandemic

A two-way ANOVA was conducted to examine the effects of gender and waves of the Coronavirus pandemic on anxiety (GAD-7), perceived stress (PSS-10), physical health (GSRH 1, and GSRH 2), and life satisfaction (SWLS). In addition, each continuous variable was compared regards three waves of the COVID-19 pandemic (W1, W2, W3) and gender (Women, Men) as factors. The results of the analysis are shown in Table 3; Fig. 4.

**Table 3:** Results of two-way ANOVA for anxiety (GAD-7), perceived stress (PSS-10), physical health (GSRH 1 and GSRH 2), and life satisfaction (SWLS) as a dependent variable, while gender and waves of the pandemic as factors

		Waves of the COVID-19 pandemic										
		W 1		W2		W3						
Variable		*M*	*SD*	*M*	*SD*	*M*	*SD*	Effect	*F*	*df*	*p*	η²_*p*_
Anxiety								W	14.94	2	< 0.001	0.015
	Women	8.98	5.45	8.51	5.54	10.25	5.90	G	68.94	1	< 0.001	0.034
	Men	7.50	5.80	6.02	5.49	7.75	5.65	WxG	1.68	2	0.190	0.002
Perceived stress								W	225.77	2	< 0.001	0.189
	Women	27.22	5.61	22.11	5.37	22.26	4.96	G	84.67	1	< 0.001	0.042
	Men	29.71	5.55	24.09	4.60	24.28	4.44	WxG	0.48	2	0.620	0.000
Physical health 1								W	26.87	2	< 0.001	0.027
	Women	2.32	0.85	2.4	0.80	2.63	0.91	G	20.42	1	< 0.001	0.010
	Men	2.47	0.74	2.56	0.75	2.82	0.82	WxG	0.1	2	0.900	0.000
Physical health 2								W	39.83	2	< 0.001	0.040
	Women	2.40	0.94	2.42	0.90	2.82	0.98	G	18.4	1	< 0.001	0.009
	Men	2.60	0.82	2.58	0.85	2.99	0.97	WxG	0.1	2	0.910	0.000
Life satisfaction								W	7.97	2	< 0.001	0.008
	Women	21.05	6.52	21.08	6.18	19.39	6.86	G	0.44	1	0.510	0.000
	Men	21.29	6.00	20.65	6.02	20.17	7.04	WxG	1.4	2	0.250	0.001

*Note.* W = Wave of the COVID-19 pandemic; G = gender; WxG = interaction between wave and gender

**Fig. 5:**
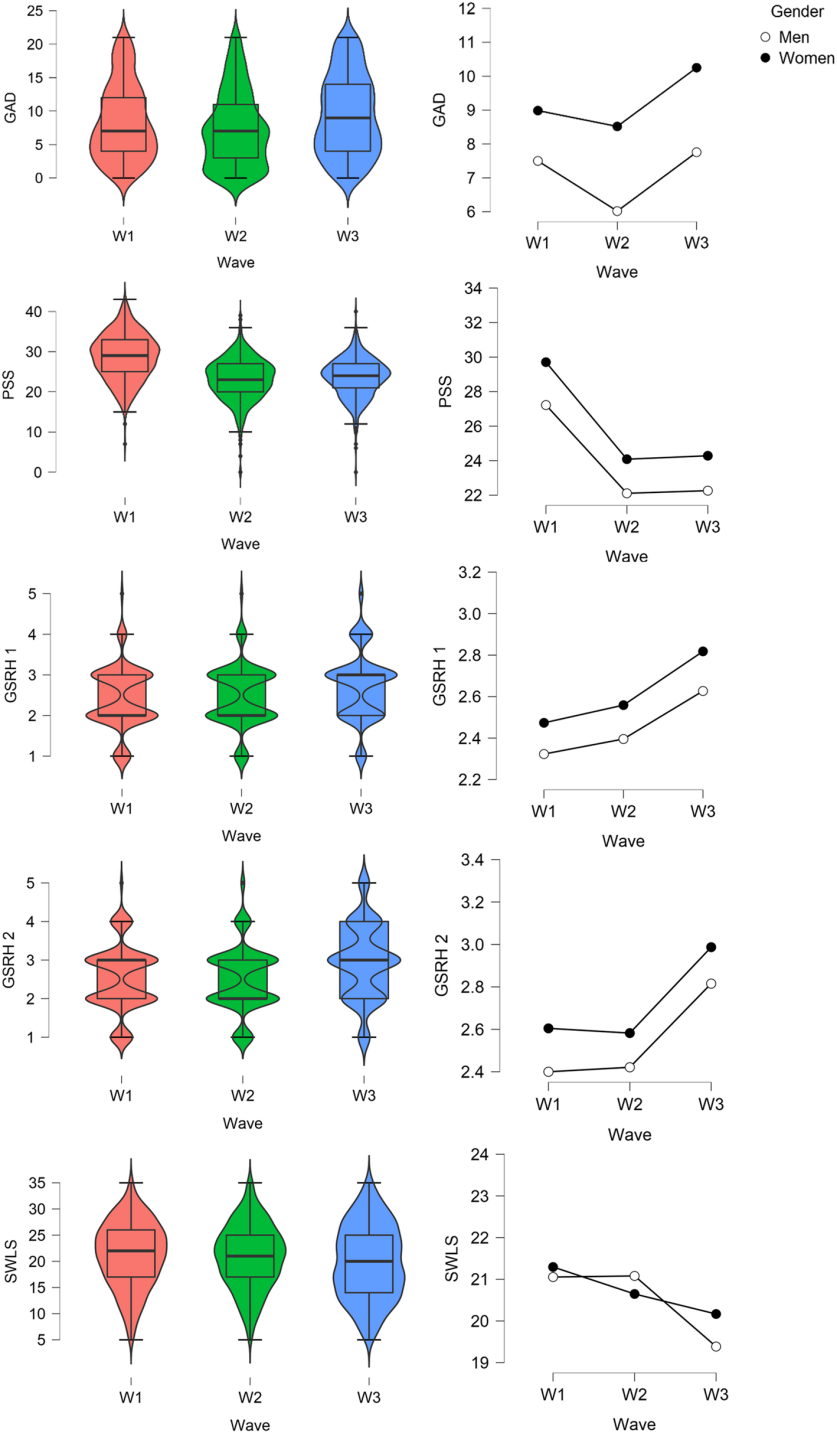
Boxplots (on the left, for the total sample) and linear plots (on the right, for gender differences), presenting scores of university students in anxiety (GAD), perceived stress (PSS), physical health (GSRH 1 and GSRH 2), and life satisfaction (SWLS) during the three waves of the COVID-19 pandemic

Significant changes in anxiety were found for pandemic wave and gender, with a small effect size for both factors (see Table 3; Fig. 5 for more details). No interaction effect between gender and pandemic wave was demonstrated in anxiety. Tuckey’s post-hoc analysis showed statistically significant differences in anxiety level between W1 and W2, with a small effect size. Higher scores at W2 were showed than at W1 (*t* = 3.09, *SE* = 0.32, *p* = 0.04, Cohen’s *d* = 0.17). Significant differences in GAD were presented between W1 and W3 (small effect size), with lower scores at W1 as compared to W3 (*t* = –2.37, *SE* = 0.32, *p* = 0.05, Cohen’s *d* = –0.13), and at W2 than W3, with small effect size (*t* = –5.44, *SE* = 0.32, *p* < 0.001, Cohen’s *d* = –0.30). Women scored significantly higher than men in anxiety (*t* = –8.30, *SE* = 0.26, *p* < 0.001, Cohen’s *d* = –0.38), with small effect size.

Considering perceived stress, statistically significant changes with large effect size were found between waves of pandemic, while gender differences were presented with small effect size (Table 3; Fig. 5). No interaction between wave and gender was shown. The Tuckey’s post-hoc tests revealed that the highest level of stress was at W1, significantly higher than at W2 (*t* = 18.86, *SE* = 0.28, *p* < 0.001, Cohen’s *d* = 0.99) and W3 (*t* = 17.88, *SE* = 0.29, *p* < 0.001, Cohen’s *d* = 0.99), with large effect size for both comparisons. However, between W2 and W3 differences were not statistically significant (*t* = –0.61, *SE* = 0.29, *p* = 0.810, Cohen’s *d* = –0.04). Women scored significantly higher than men in perceived stress (*t* = –9.2, *SE* = 0.23, *p* < 0.001, Cohen’s *d* = –0.38), with small effect size.

A significant main effect of wave and gender (but not interaction) was presented for physical health, considering both questions GSRH 1 and GSRH 2, although effect size was small (Table 3; Fig. 5). The Tuckey’s post-hoc tests showed that significant changes in GSRH 1 are between W1 and W3 (*t* = –7.04, *SE* = 0.05, *p* < 0.001, Cohen’s *d* = –0.39), and also between W1 and W3 in GSRH 2 (*t* = –7.73, *SE* = 0.05, *p* < 0.001, Cohen’s *d* = –0.43), but with a small effect size. Similarly, significantly worst physical health was reported at W3 than W2 regarding GSRH 1(*t* = –5.37, *SE* = 0.05, *p* < 0.001, Cohen’s *d* = –0.30), as well as GSRH 2 (*t* = –7.80, *SE* = 0.05, *p* < 0.001, Cohen’s *d* = –0.43), however effect size was small. Women felt physically worst that men, with small effect size, considering both GSRH 1 (*t* = –4.52, *SE* = 0.04, *p* < 0.001, Cohen’s *d* = –0.21) and GSRH 2 (*t* = –4.29, *SE* = 0.04, *p* < 0.001, Cohen’s *d* = –0.19).

Life satisfaction changed by wave, but significant main effect was not found for gender or interaction between wave and gender (Table 3; Fig. 5). The Tuckey’s post-hoc tests showed significant differences between W1 and W3 (*t* = 3.81, *SE* = 0.37, *p* < 0.001, Cohen’s *d* = 0.21), and between W2 and W3 (*t* = 2.99, *SE* = 0.36, *p* = 0.001, Cohen’s *d* = 0.17), but not between W1 and W2 (*t* = 0.86, *SE* = 0.36, *p* = 0.66, Cohen’s *d* = 0.05).

### Predictors of anxiety in university students

The logistic regression was performed to find predictors of GAD risk in university students (Table 4). Almost all predictor variables were significant for anxiety, including the second wave (as compared to W3), female gender, high perceived stress, both items of poor physical health (GSRH 1 and GRSH 2), and low level of life satisfaction. Anxiety was around two times more frequent in the second wave of the COVID-19 pandemic and among women and people who self-rated their health as fair or poor. Anxiety was three times more frequent among participants dissatisfied with their life, and seven times more likely among students with high-stress levels. The model explains one-third of anxiety variation, Cox and Snell’s *R*^2^ = 0.26, Nagelkerke’s *R*^2^ = 0.36.

**Table 4:** Logistic regression for anxiety (*N* = 1961)

				Wald’s χ^2^				95% *CI*	
Variable		*b*	*SE b*		*df*	*p*	*AOR*	*LL*	*UL*
Constant		-3.20	0.18	306.74	1	< 0.001	0.04		
Wave				19.17	2	< 0.001			
	W1 vs. W3	0.18	0.14	1.67	1	0.196	1.19	0.91	1.56
	W2 vs. W3	0.59	0.14	18.31	1	< 0.001	1.81	1.38	2.38
Gender (Women)		0.65	0.12	31.43	1	< 0.001	1.91	1.52	2.39
Perceived stress (High)		2.00	0.14	215.90	1	< 0.001	7.40	5.67	9.67
Physical health									
	GSRH 1 (Worse)	0.90	0.23	15.30	1	< 0.001	2.46	1.57	3.87
	GSRH 2 (Worse)	0.64	0.18	12.45	1	< 0.001	1.89	1.33	2.70
Life satisfaction (Dissatisfy)		1.10	0.11	95.64	1	< 0.001	3.02	2.42	3.76

*Note*. GSRH = General Self-rated Health; *CI* = confidence interval; *LL* = lower level; UL = upper level

## Discussion

### Changes in mental health in comparison of the three pandemic waves

This study aims to compare the prevalence of mental health problems across three waves of the COVID-19 pandemic. Although anxiety slightly decreases during W2 compared to W1, it significantly increases in W3 during the pandemic. In contrast, perceived stress was higher at W1 and significantly decreased in the following waves of the COVID-19 pandemic (W2 and W3). University students felt worse and worse in the successive waves of the pandemic (GSRH 1), and much worse at W3 than W1 and W2 when they compared self-health to the people at the same age (GSRH 2). Also, life satisfaction has declined in the following waves of the pandemic. Female gender was a significant risk factor for anxiety, perceived stress, and physical health (particularly when self-health was compared to others at the same age), but the effect size was small. High stress and low life satisfaction are the best predictors of high GAD risk. However, high anxiety was also two times more frequent among women and people with poor self-rated health.

High stress and a moderate level of anxiety at W1 seem consistent with a previous study conducted at the beginning of the pandemic by Bareequa et al. [11]. A systematic review and meta-analysis, performed during the early period of the COVID-19 crisis in China, showed a very high prevalence of stress (48.1%) and a moderately high prevalence of anxiety. The pooled prevalence of anxiety was 21.8% among the Chinese population, although the prevalence of 29.2% was found using the GAD-7 [11]. The highest prevalence of anxiety (38.12%) was found by Necho et al. [13], but it differed depending on the.geographical region or a measurement instrument. In general, there are vast disparities in the prevalence of mental health issues between review studies Previous research found in the general population that the prevalence of anxiety ranged between 15.15% and 38.12%, while the prevalence of high stress ranged between 13.29% and 48.1% [11–14, 16, 17, 19]. Similarly, research reports differ in the prevalence of mental health problems among university students [8, 20, 23–27, 33–39]. Perceived stress ranged between 24.7% and 71.23% [20, 25, 28, 37], while anxiety between 11.0% and 51.68% [8, 20, 25, 28, 37] in various studies.

Lifestyle and mental health were disrupted during COVID-19, as indicated by Giuntella et al. [9]. College and university students experienced many negative changes in the online educational system, which affected academic and social lives, increasing financial problems, lack of social relationships and sense of belonging, housing and food insecurity, and a high degree of uncertainty about the future [50]. The negative impact of the COVID-19 pandemic in several fields of students’ life was associated with higher stress and poorer self-rated health [10, 51]. Research indicates that the high levels of anxiety and frustration among university and college students were associated with greater concern about school-related problems, such as worry about insufficient computer skills, poor quality of online classes, passing classes and exams online, academic performance, professional career opportunities, and future studies [52–54]. De la Fuente et al. [6] showed that stress factors from the teaching process were related to the learning process and academic burnout. The previous and current research indicates the need to develop prevention and intervention programs at campuses, which could focus on improving computer and online-learning-related skills and reduce stress and anxiety.

Physical health and life satisfaction were in the lower interests of scientists during the COVID-19 pandemic. We found in the present study that a small percent of university students self-rated their health as fair or poor (10.15%), but slightly greater percent (16.42%) when they compared their health to others at the same age. Significant but weak differences were found in self-rating general health, with an increasing proportion of people poorly rating their health from W1 (6.54%) to W3 (16.92%). Similar results were found in the first wave of the pandemic in Poland, with 6% of people with poor health status [25]. Among participants in the study, 42.33% reported dissatisfy with their life, and the frequency of dissatisfied people grew with the following waves, up to almost half in W3 (49.54%). The present results are consistent with previous research, which showed significant decreases in wellbeing and life satisfaction among university students during the COVID-19 pandemic [20, 25, 27, 33, 38, 39]. For example, people dissatisfied with their lives were 32% in Poland [25], but as high as 56.42% in Turkey [20] during the early pandemic time.

Nochaiwong et al. [14] found in their systematic review and meta-analysis that 30.7% of people worldwide complained of somatic symptoms during the COVID-19 pandemic, and 28.6% reported low wellbeing. More research from the whole globe should compare the prevalence in subsequent waves of the pandemic to thoroughly compare the present results with the other studies. Deng et al. [8] found significant differences regarding geographical regions, diagnostic criteria, education level, undergraduate year of study, financial situation, living arrangements, and gender. Future studies could take into consideration more demographic variables to explain disparities between particular studies. Deng et al. [8] suggest that mental health screening and intervention should be a top priority for universities and colleges during the pandemic. Indeed, the present research and previous review studies show that society worldwide must develop surveillance, prevention, and intervention programs during and after this worldwide crisis [12].

### Gender differences

The study indicated that women are at higher risk of anxiety (45.52% vs. 27.98%) and stress (62.21% vs. 51.66%) than men. Also, women worse self-rated their physical health if they compared it to other people at the same age. No gender differences were found in physical health and life satisfaction for categorical data. Nevertheless, when the analysis was performed for mental health variables considered as continuous, women showed significantly higher scores in anxiety and perceived stress, and they felt physically worst than men. No gender differences were found in life satisfaction as a continuous variable. It is important to note, however, that the effect size was weak for gender differences in Pearson’s χ^2^ test as well as for ANOVA.

The study results are consistent with previous studies that also found gender differences in mental health problems [8, 11, 20, 30, 52] A systematic review and meta-analysis for studies performing during the COVID-19 pandemic showed that anxiety prevalence is higher in females than in the general Chinese population [11]. The pooled prevalence of anxiety in 21,391 females from China was 25% [11]. Female gender was also a risk factor for mental health among French university students [28]. However, recent findings suggest that the mental health of populations around the world during the COVID-19 pandemic does not differ across affected countries or gender [12]. More research is necessary to verify the present and previous results.

### Predictors of anxiety

Results of this study indicate that although all variables are significant for explaining anxiety variation, perceived stress is the most important during the pandemic crisis. University students with high levels of perceived stress were above seven times more likely to indicate high anxiety disorder risk. A review by O’Connor et al. [55] showed that stress could affect health directly and indirectly via changes in health behaviors, which may lead to various adverse mental and physical health outcomes, including a high risk of infectious diseases (such as the COVID-19). Also, a low level of life satisfaction was found as a predictor of high anxiety. Life satisfaction is related to several factors, including income, job satisfaction, needs satisfaction, resilience, physical health, and social relationships and support. In contrast, unemployment, or high levels of stress, anxiety, and depression, has decreased life satisfaction [56–59]. Furthermore, gender and third wave of pandemic were significant predictors of high anxiety level. The COVID-19 pandemic disrupted almost all areas of human life, including school, work, family, and social life, which caused high stress in global populations. Therefore, prevention and intervention programs during the COVID-19 pandemic should target high levels of perceived stress and anxiety to help them improve coping skills and teach new methods to manage stress and anxiety.

### Study limitations

Although this study identified significant strong evidence of changes in mental health during the COVID-19 pandemic in a large sample of university students, the findings should be interpreted with caution due to the cross-sectional cohort design. The three samples were not matched and differred in many demographic or personal characteristics. Further research should be aimed to perform longitudinal repeated measures study for matched samples. Although subsequent surveys were completed after the six-month break, some students may have remembered previous responses, so a recall bias may occur. University students represented various types of universities and hundreds of study majors, but the online method of recruiting participants to gain a convenience sample using social media and e-mailing a list of students does not allow us to generalize the results of this study to the population of university students as a whole. In particular, convenience sample can lead to selection bias. Also, the present research includes university students from Poland. A cross-cultural study could be conducted to compare the present results with other samples from various countries and geographic regions of the world. Self-reported measures included in a survey may also be a source of potential bias.

## Conclusions

The results of the study are consistent if comparing mental health variables considered as categorical and continuous. Significant changes across gender and three waves of the COVID-19 pandemic were found in anxiety, perceived stress, self-reported physical health, and life satisfaction. It means that future systematic reviews and meta-analyses could be more sensitive at the period of data collecting in particular studies. Wave of pandemic, as well as gender, should be consider in the future studies as factors affecting mental health condition. The development and implementation of adequate prevention and intervention programs at universities should be a priority in the fight against the COVID-19 pandemic.

## Data Availability

The datasets used and analyzed during the current study are available from the corresponding author on reasonable request.

## Abbreviations

ANOVA: analysis of variance
COVID-19: Coronavirus Disease 2019
GAD: general anxiety disorder
GAD-7: seven-items General Anxiety Disorder scale
GSRH: General Self-Rated Health
PSS-10: ten-items Perceived Stress Scale
SPSS: Statistical Packages for the Social Sciences
SWLS: Satisfaction with Life Scale
